# Iatrogenic Hypoglycemia in Type 2 Diabetes Affects Endothelial Proteins Involved in Cardiovascular Dysfunction

**DOI:** 10.3390/ijms27020822

**Published:** 2026-01-14

**Authors:** Edwina Brennan, Abu Saleh Md Moin, Thozhukat Sathyapalan, Laura Dempsey, Stephen L. Atkin, Alexandra E. Butler

**Affiliations:** 1 School of Medicine, Royal College of Surgeons in Ireland Medical University of Bahrain, Busaiteen P.O. Box 15503, Bahrain; 2 School of Postgraduate Studies and Research, Royal College of Surgeons in Ireland Medical University of Bahrain, Busaiteen P.O. Box 15503, Bahrain; amoin@rcsi.com (A.S.M.M.); satkin@rcsi.com (S.L.A.); abutler@rcsi.com (A.E.B.); 3 Academic Endocrinology, Diabetes and Metabolism, Hull York Medical School, Hull HU6 7RX, UK; thozhukat.sathyapalan@hyms.ac.uk; 4 Data Science Centre, School of Population Health, Royal College of Surgeons in Ireland, D02 DH60 Dublin, Ireland; lauradempsey@rcsi.ie

**Keywords:** hypoglycemia, type 2 diabetes, T2D, endothelial proteins, selectins, plasminogen activator inhibitor-1, intercellular adhesion molecule 3

## Abstract

Hypoglycemia is associated with cardiovascular events reflected by platelet abnormalities. We hypothesized that sequential endothelial changes may occur during hypoglycemia that may enhance cardiovascular risk. In type 2 diabetes (T2D) (*n* = 23) and controls (*n* = 23), blood SOMAscan proteomic analysis of endothelial proteins at baseline, insulin-induced hypoglycemia and post hypoglycemia to 24 h were examined using repeated-measures linear mixed modeling with a prospective parallel study design. Most endothelial proteins that changed over time did not differ between groups. Baseline levels of P-selectin, plasminogen activator inhibitor-1 (PAI-1; serpine-1), E-selectin and angiopoietin-1 (ANGPT1) were significantly higher, whilst cadherin-5 was lower in T2D. Several proteins exhibited changes versus baseline in both T2D and controls. Under hypoglycemia, decreases in cadherin-5 and soluble angiopoietin-1 receptor (sTie-2) were observed, with increased P-selectin, intercellular adhesion molecule-3 (ICAM3), ANGPT1 and PAI-1. Post hypoglycemia, decreased cadherin-5 and ICAM5 were observed at 2 h and PAI-1 at 4 h, as well as increases in P-selectin at 30 min, 1 h and 24 h and ICAM3 at 24 h. Post hypoglycemia, E-selectin, P-selectin and ICAM3 were significantly lower in T2D patients at 2 h, while PAI-1 was significantly lower at 4 h and ICAM3 was significantly lower at 24 h. Baseline endothelial proteins differed between T2D and controls, which may suggest local endothelial inflammatory activation leading to a pro-thrombotic, destabilized vascular phenotype characteristic of diabetic vasculopathy. Hypoglycemia may exacerbate this towards a pro-adhesive and pro-thrombotic phenotype, worsening endothelial dysfunction.

## 1. Introduction

Treatment of patients with type 2 diabetes (T2D) with insulin or sulphonylurea therapy in an intensive therapeutic regime may lead to hypoglycemia, which may result in loss of consciousness, seizure, coma and even death [1]. T2D is characterized by prolonged hyperglycemia and insulin resistance, often in conjunction with obesity, dyslipidemia and hypertension. T2D is associated with cardiovascular disease (CVD) [2], with individuals with T2D experiencing more than a two-fold higher risk than those without diabetes [3]. Additionally, clinical trials of glucose-lowering interventions in T2D report associations between hypoglycemia and risk factors for cardiovascular outcomes [4,5], while subsequent trial follow-ups report significant associations with increased risk of cardiovascular events (macro- and microvascular) and cardiovascular-related death [6,7,8]. Moreover, [9] reports that patients with T2D experiencing hypoglycemia are at greater risk of subsequent hypoglycemia, as well as major adverse cardiovascular events.

Multiple inter-related pathophysiological pathways are implicated in the development of cardiovascular dysfunction in T2D: oxidative stress, inflammation, fibrosis, lipotoxicity and excitation–contraction disruption [10]. Hypoglycemic assault imposes additional stress that further disrupts these pathways [11]. T2D is considered a prothrombotic state characterized by enhanced coagulation [12], impaired fibrinolysis via elevated plasminogen activator inhibitor-1 (PAI-1) levels [13] and platelet hyperactivity [14]. Early endothelial dysfunction, oxidative stress and vascular inflammation promote monocyte recruitment and macrophage dysfunction, leading to foam-cell formation and eventual atherosclerotic plaques [15]. Hypoglycemia in T2D patients has been shown to further exacerbate the condition through prothrombotic effects [16], enhanced platelet activation [17,18], inflammatory responses [19,20], altered endothelial microparticles [21] and cardiac arrhythmias [22] and to attenuate the vascular endothelial protective effect of glucagon-like peptide-1 (GLP-1) therapy [23]. While hypoglycemia stimulates counter-regulatory hormones such as epinephrine and glucagon to restore euglycemia, in those with diabetes, such counter-regulatory responses are impaired and are negatively impacted by recurrent episodes [24].

A healthy endothelium responds to stimuli by producing various factors that regulate vascular tone, cellular adhesion, thromboresistance, smooth-muscle-cell proliferation and vessel-wall inflammation. Endothelial dysfunction is fundamental to alterations in vascular processes and is an early indicator of the development of atherosclerosis [25]. Mechanisms involved include nitric oxide availability, oxidative stress and inflammation, which lead to a cascade of effects: endothelial permeability, platelet aggregation, leukocyte adhesion and the generation of cytokines [25]. While markers of endothelial dysfunction such as acute-phase inflammatory proteins, cytokines and cellular adhesion molecules have been assessed as early indicators of cardiovascular risk [26], investigation under hypoglycemic conditions in T2D is under-reported. Therefore, this study was undertaken to determine whether changes in endothelial proteins following hypoglycemia may involve protein markers of endothelial activation/dysfunction, vascular integrity, inflammation, coagulation and fibrinolysis, and endothelial progenitor cell-related proteins in adults with and without T2D, with the null hypothesis that endothelial proteins do not differ in their response between the two groups.

## 2. Results

### 2.1. Demographic and Biochemical Characteristics of Study Participants

T2D patients were significantly older (64 versus 60 years, *p* < 0.05) and had higher hemoglobinA1c (HbA1c) (6.8 versus 5.6%, *p* < 0.0001), body weight (90.9 versus 79.5 kg, *p* < 0.0001) and body mass index (BMI) (32 versus 28 kg/m^2^, *p* < 0.0001) values compared to controls (Table 1) [27]. Systolic blood pressure (BP) and diastolic BP were higher in T2D compared to controls: 132 versus 122 mmHg (*p* = 0.001) and 81 versus 75 mmHg (*p* = 0.003), respectively. T2D patients had significantly lower total cholesterol (4.2 versus 4.8 mmol/L, *p* = 0.014) and high-density lipoprotein (HDL) cholesterol (1.1 versus 1.5 mmol/L, *p* = 0.001), while triglycerides and low-density lipoprotein (LDL) cholesterol levels were similar (*p* > 0.05). C-reactive protein (CRP), as a measure of inflammation, did not differ (*p* > 0.05).

### 2.2. Comparison Between T2D and Controls at Baseline

**Figure 1:**
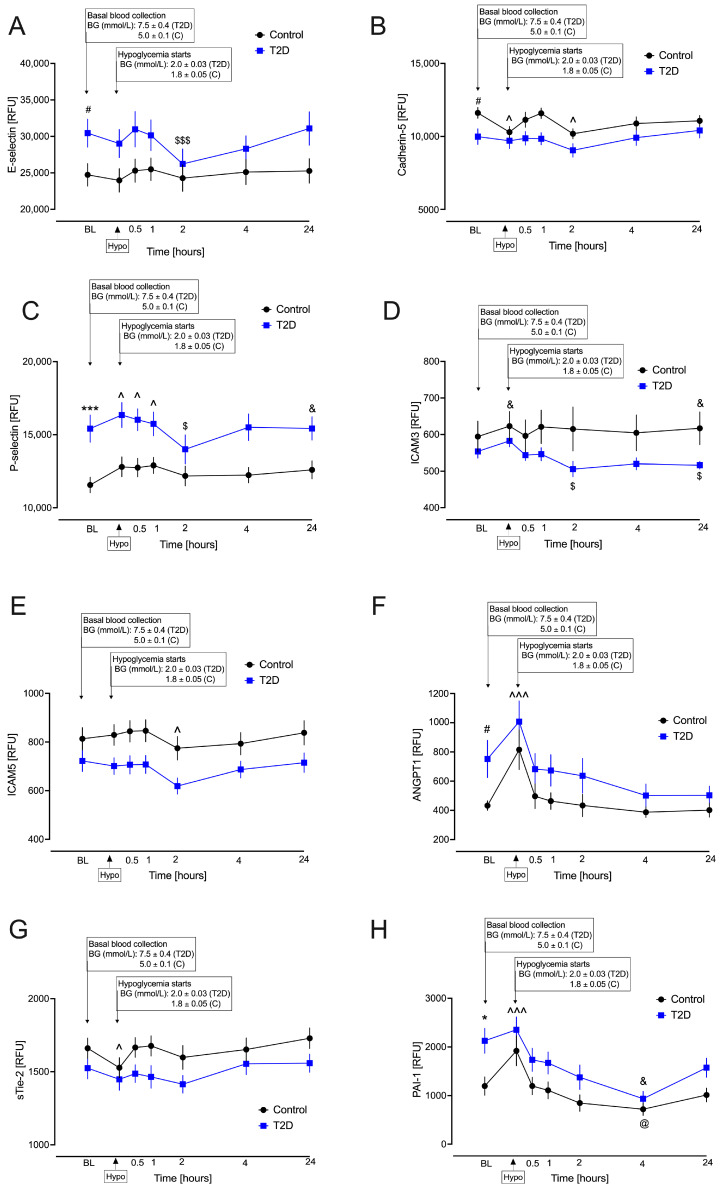
Significant changes in endothelial activation/dysfunction markers in plasma before, during and after iatrogenic induction of hypoglycemia. Blood sampling was performed at baseline (BL), under hypoglycemia (0 min) and post hypoglycemia (30 min and 1, 2, 4 and 24 h) for controls (black circles) and for type 2 diabetes (T2D) (blue squares). At BL, blood sugar (BS) was 7.5 ± 0.4 mM (for T2D) and 5.0 ± 0.1 mM (for control, C). At the point of hypoglycemia, blood glucose (BG) was 2.0 ± 0.03 mM (for T2D) and 1.8 ± 0.05 mM (for control). (**A**) E-selectin; (**B**) Cadherin-5; (**C**) P-selectin; (**D**) intercellular adhesion molecule-3 (ICAM3); (**E**) ICAM5; (**F**) angiopoietin-1 (ANGPT1); (**G**) soluble angiopoietin-1 receptor (sTie-2); (**H**) plasminogen activator inhibitor-1 (PAI-1; serpine 1). Data are presented as mean ± SEM. Statistics: # *p* < 0.05, * *p* < 0.01 and *** *p* < 0.0001 for comparison between T2D and controls at BL; @ *p* < 0.05, ^$^ *p* < 0.01 and ^$$$^ *p* < 0.0001 for comparison between T2D and controls under hypoglycemia (0 min) and post hypoglycemia (30 min and 1, 2, 4 and 24 h); ^&^ *p* < 0.05, ^ *p* < 0.01 and ^^^ *p* < 0.0001 for comparison between BL for the combined cohort, hypoglycemia (0 min) and post hypoglycemia (30 min and 1, 2, 4 and 24 h).

At baseline, T2D had significantly elevated E-selectin (*p* = 0.03) (Figure 1A), P-selectin (*p* < 0.0001) (Figure 1C), PAI-1 (*p* < 0.01) (Figure 1H) and angiopoietin-1 (ANGPT1) (*p* = 0.02) (Figure 1F), and lower cadherin-5 (*p* = 0.02) (Figure 1B) compared to healthy control subjects. There were no significant differences (*p* > 0.05) in baseline levels of the remaining biomarkers (Supplemental Figures S1 and S2). Cohen’s *d* showed that there were large effect sizes for P-selectin and PAI-1 and medium effect sizes for E-selectin, cadherin-5 and ANGPT1, whilst intercellular adhesion molecules (ICAM)-3 and -5 and soluble angiopoietin-1 receptor (sTie-2) showed small effect sizes and did not differ between T2D and controls at baseline (Table 2). When the false discovery rate (FDR) was corrected, only P-selectin (*p* = 0.017) and PAI-1 (*p* = 0.045) remained significant.

### 2.3. Changes in Endothelial Biomarkers

The approach of using a linear mixed model for repeated measures allows us to evaluate both overall trends (combined groups) and group-specific effects. In most instances, it was observed that the way in which endothelial markers changed over time did not differ between groups.

#### 2.3.1. Comparison Between T2D and Controls Under Hypoglycemia and Post Hypoglycemia

At the point of induced hypoglycemia, there were no significant differences for any of the measured biomarkers between T2D and healthy control subjects.

During the post-hypoglycemic follow-up, T2D subjects had significantly reduced levels of E-selectin (*p* < 0.001) (Figure 1A), P-selectin (*p* < 0.01) (Figure 1C) and ICAM3 (*p* < 0.01) (Figure 1D) at 2 h, PAI-1 (*p* = 0.04) (Figure 1H) at 4 h and ICAM3 (*p* < 0.01) (Figure 1D) at 24 h compared to controls. There were no significant interaction effects (*p* > 0.05) observed for the remaining measured biomarkers (Supplemental Figures S1 and S2) post hypoglycemia.

#### 2.3.2. Comparison Between Baseline Values for the Combined Cohort Under Hypoglycemia and Post Hypoglycemia

In the combined cohort, upon induction of hypoglycemia, there were significant decreases in cadherin-5 (*p* = 0.01) (Figure 1B) and sTie-2 (*p* = 0.01) (Figure 1G) and significant increases in P-selectin (*p* < 0.01) (Figure 1C), PAI-1 (*p* < 0.001) (Figure 1H), ANGPT1 (*p* < 0.001) (Figure 1F) and ICAM3 (*p* = 0.03) (Figure 1D) compared to baseline. There were no significant differences (*p* > 0.05) in the remaining measured endothelial activation/dysfunction, vascular integrity, inflammation, coagulation and endothelial progenitor biomarkers under hypoglycemia compared to baseline in the combined cohort (Supplemental Figures S1 and S2).

In the combined cohort, there were significant decreases in cadherin-5 (*p* = 0.01) (Figure 1B) and ICAM5 (*p* < 0.01) (Figure 1E) at 2 h and in PAI-1 (*p* = 0.02) (Figure 1H) at 4 h post hypoglycemia in both T2D and controls compared to baseline. Post hypoglycemia, P-selectin was significantly increased at 30 min (*p* = 0.01), 1 h (*p* = 0.01) and 24 h (*p* = 0.03), and ICAM3 was significantly increased at 24 (*p* = 0.047) in both T2D and controls compared to baseline (Figure 1C and Figure 1D, respectively). Two-hour glucose was higher in T2D compared to controls (9.9 ± 1.3 vs. 6.9 ± 0.5mmol/L, *p* = 1.8 × 10^−11^), but there was no correlation between the 2 h glucose and the protein parameters that changed in either controls or T2D.

There were no significant interaction effects, indicating a lack of evidence that the way the two groups changed over time differed for ICAM1 and 2, vascular cell adhesion molecule-1 (VCAM1), angiopoietin 2 (ANGPT2), interleukin (IL) 1, IL6, D-dimer, stromal cell-derived factor-1 (SDF-1), von Willebrand factor (vWF), vascular endothelial growth factor (VEFGA), tumor necrosis factor alpha (TNFα), tissue plasminogen activator (tPA) or tissue factor (TF) (Supplemental Figures S1 and S2, Supplementary Table S1).

The Framingham 10-year General CVD risk with T2D status included is shown in Table 1, showing that the diabetes group has a significantly higher Framingham 10-year CVD risk than controls, with an effect size (d ≈ 1.7) indicating a very large between-group separation. Correlations within each group separately (N = 23 per group) showed no significant correlations for any of the baseline proteins with Framingham risk for either T2D or controls. However, vWF demonstrated a consistent inverse trend with Framingham risk in both controls (Pearson r = −0.35, *p* = 0.10) and T2D subjects (r = −0.34, *p* = 0.11). In parallel, linear regression models testing differential associations by group (protein ~ Framingham risk + group + Framingham x group; N = 46) identified no significant Framingham x group interactions, indicating that the slope relating Framingham risk to each protein did not differ detectably between controls and T2D subjects.

Platelet counts were numerically higher in the T2D group (Table 1), but the difference did not reach statistical significance using an independent t-test; however, the effect size (d ≈ 0.46) suggested a modest increase in platelets in diabetes. Within-group correlation analyses demonstrated no statistically significant associations between platelet count and circulating endothelial proteins after FDR correction in either controls or T2D. In the T2D group, platelet count showed a modest positive trend with P-selectin (r = 0.11, *p* = 0.08), whereas correlations were weaker and inconsistent in controls.

### 2.4. Protein–Protein Interaction Network

**Figure 2:**
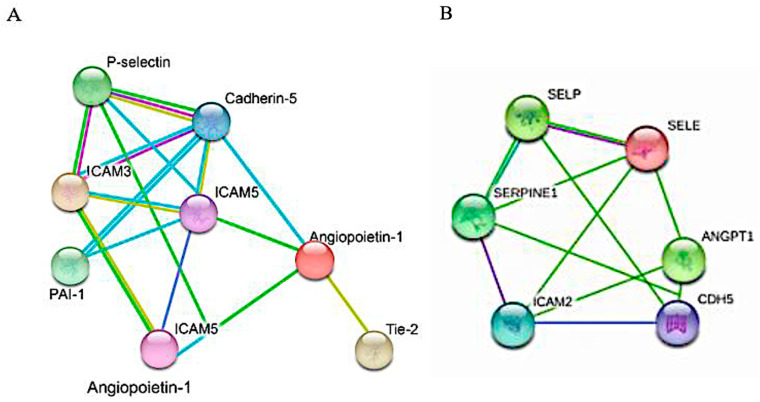
STRING version 12.0 interaction network was used to generate protein–protein interactions between the proteins measured by the SOMAscan assay. (**A**) All proteins included in the study that showed changes either at baseline or during the hypoglycemia time course. (**B**) Dysregulated proteins that differed between T2D and controls at baseline. SELP = p-selectin; SELE = E selectin; ANGPT1 = angiopoietin-1; ICAM = intercellular adhesion molecule-1; SERPINE1 = PAI 1. Darker green lines indicate neighborhood proteins; purple lines indicate gene fusion co-occurrence; light blue lines indicate co-expression determined experimentally; lime green lines indicate interactions determined from curated databases.

The STRING protein–protein interaction network between the proteins measured by the SOMAscan assay revealed close associations between proteins. All proteins that differed at baseline and/or during the hypoglycemia time course are visualized in Figure 2A, whilst only the proteins that differed between T2D and controls at baseline are shown in Figure 2B. Gene ontology (GO) and pathway synthesis showed three tightly coupled functional axes—namely, E-selectin, ICAM3 and ICAM5 for endothelial activation and leukocyte adhesion; P-selectin and vWF for platelet adhesion; and ANGPT1 and PAI-1 for angiopoietin fibrinolytic balance. GO enrichment for biological processes showed cell adhesion with E-selectin, P-selectin, ICAM3 and ICAM5; leukocyte migration with E-selectin, P-selectin and ICAM3; and blood coagulation and hemostasis with E-selectin and PAI-1. GO enrichment for molecular function showed E-selectin, ICAM3 and ICAM5 to be associated with cytokine activity. GO enrichment for cellular components showed cell-surface interaction for E-selectin, P-selectin, ICAM3 and ICAM5.

## 3. Discussion

This study examines endothelial proteins in T2D and control groups using a repeated-measures linear mixed model at baseline and following iatrogenic hypoglycemia for a period of 24 h. We observed significant baseline differences between groups for several proteins with higher E-selectin, P-selectin, ANGPT1 and PAI-1 and reduced cadherin-5 in T2D, which may suggest inflammatory endothelial activation leading to a pro-thrombotic, destabilized vascular phenotype characteristic of diabetic vasculopathy.

Several proteins exhibited significant changes over time compared to baseline, particularly under hypoglycemia (cadherin-5 and sTie-2 showed decreases, while P-selectin, ICAM3, ANGPT1 and PAI-1 showed increases compared to baseline) and in the subsequent hours post hypoglycemia (an increase at 30 min, 1 h and 24 h for P-selectin; a decrease at 2 h for ICAM5; a decrease at 4 h for PAI-1; an increase at 24 h for ICAM3; and a decrease at 2 h for Cadherin-5). The most notable findings in terms of differences between the diabetes and control groups over time include significant decreases in E-selectin, ICAM3 and P-selectin at 2 h post hypoglycemia in the T2D group compared to the control group, as well as decreases in PAI-1 at 4 h and ICAM3 at 24 h post hypoglycemia in the T2D group compared to the control group. Overall, cadherin-5 and sTie-2, together with increases in P-selectin, ICAM3, ANGPT1 and PAI-1, suggest that hypoglycemia may result in a shift from endothelial stability to an inflammatory, pro-adhesive and pro-thrombotic phenotype, indicating worsening endothelial dysfunction after baseline. In addition, there were increases in both P-selectin and ICAM3 that were maintained at 24 h, suggesting platelet–endothelial activation in accordance with potentially persistent pro-inflammatory and pro-thrombotic vascular changes after hypoglycemia.

P-selectin and E-selectin are endothelial/platelet adhesion molecules that mediate leukocyte rolling and adhesion under flow conditions. Increased E-selectin and P-selectin levels reflect a prothrombotic state characteristic of T2D [12] and platelet hyperactivity. E-selectin is uniquely expressed by endothelial cells and induced by TNF-α, IL-1β and lipopolysaccharide, while P-selectin is also produced by platelets, redistributing to the surface via thrombin, histamine and complement proteins [28]. Selectins are implicated as playing a role in thrombosis via tissue-factor expression [29]. In fact, E-selectin levels are associated with severe post-thrombotic syndrome [30], while inhibition decreases thrombosis and vascular fibrosis [31]. In addition, P-selectin promotes large platelet aggregation by stabilizing glycoprotein–fibrinogen interactions and is strongly expressed by endothelia with active atherosclerotic plaques [32]. In this study, significant decreases in E-selectin and P-selectin at 2 h post hypoglycemia were observed in T2D compared to controls. Whether this reduction infers a greater protective effect in those with T2D against the prior prothrombotic state or suggests enhanced risk of destabilized blood clots leading to potential emboli requires further study. In the combined cohort, higher P-selectin levels were observed under hypoglycemia and post hypoglycemia (30-min, 1 h and 24 h). This elevation in P-selectin up to 24 h post hypoglycemia is consistent with flow cytometry studies [16,17,33] and serum levels [34] in other T2D cohorts. Increases in E-selectin in both T2D and controls reported by others [34] and higher peak levels of E-selectin microparticles within 240 min in T2D [21] were not observed in this study.

In addition to its role as a cell adhesion molecule, ICAM3 promotes macrophage chemoattraction to sites of leukocyte cell death and subsequent leukocyte tethering [35]. ICAM3 significantly increased under hypoglycemia and at 24 h post hypoglycemia in the combined cohort, but compared to controls, T2D subjects had decreased levels at 2 and 24 h post hypoglycemia, suggesting endothelial dysfunction that differs between those with T2D and those without T2D.

ICAM5 is expressed exclusively within the telencephalon and is suggested to play a role in the formation, maintenance and plasticity of neuronal networks [36]. In an in vitro ischemic stroke model, hippocampal cells were shown to express ICAM5 [37]. The reduced levels of ICAM5 in the combined cohort from baseline to 2 h post hypoglycemia may reflect impaired neuroprotection and cognitive function, consistent with hypoglycemia in those with and without diabetes [20].

Cadherin-5, expressed at intercellular junctions of endothelial cells, plays a pivotal role in vascular integrity and permeability. Plasma exosomes from patients with coronary artery disease were found to suppress cadherin-5 in human aortic endothelial cells, negatively impacting vascular permeability and causing aggravated atherosclerotic lesions [38]. In this study, cadherin-5 levels were lower in T2D subjects compared to controls at baseline, suggesting dysregulated vascular integrity in this group. To the best of our knowledge, no previous human study has examined cadherin-5 levels following a hypoglycemic event, but an in vitro endothelial hypoglycemic model found altered disruption and relocation mediated by Nuclear Factor Erythroid 2-Related Factor 2 (Nrf2) signaling [39]. Consistent with that study, in the combined cohort, cadherin-5 levels decreased under hypoglycemia and 2 h post hypoglycemia, inferring transient impacts on vascular integrity.

In this study, hypoglycemia did not result in changes in VEGFA, ICAM1, ICAM2 and VCAM1 as known mediators of endothelial dysfunction associated with atherosclerosis, stroke and myocardial infarction [40,41,42], as well as T2D microvascular complications [43]. Others reported increased levels of VEFGA, ICAM1 and VCAM1 50 min post hypoglycemia (3.2 mmol/L) in T2D [34]. Our results are in accordance with others who showed no changes in ICAM1 and VCAM1 post hypoglycemia [33].

PAI-1 (Serpine-1) is a key regulator of fibrinolysis but also links into pathways of endothelial dysfunction, vascular remodeling and inflammation. It is produced by endothelial cells, platelets and adipose tissue, inhibiting the conversion of plasminogen to plasmin by tPA and urokinase-type plasminogen activator (u-PA) [44]. PAI-1 is a risk factor for atherothrombotic events and is overexpressed in T2D atherosclerotic plaques [13]. The higher PAI-1 at baseline in the T2D group suggests a prothrombic state. Decreases in PAI-1 4 h post hypoglycemia in the T2D group are in accordance with results reported by others [34]. In the combined cohort, PAI-1 levels increased under hypoglycemia and decreased following hypoglycemia, in accordance with results reported by others [16,33].

ANGPT1 is a glycoprotein ligand that interacts with angiopoietin-1 receptor (Tie-2), which is expressed exclusively on endothelial cells. Endothelial shedding of cellular Tie-2 yields sTie-2, which circulates in plasma and serum. In vitro, sTie-2, induced by VEGF and mediated by the phosphatidylinositol 3-kinase (PI3K)/protein kinase B (Akt) and p38 mitogen-activated protein kinase (p38 MAPK) pathway has been shown to bind to ANGPT1, inhibiting ANGPT1-mediated Tie-2 phosphorylation and anti-apoptosis [45], thereby providing vascular protective effects. In this study, levels of ANGPT1 were higher at baseline in T2D subjects compared to controls, consistent with its previously reported protective effects against renal disease in those with diabetes [46], while sTie-2 levels were similar. At the point of hypoglycemia, levels of ANGPT1 increased, while sTie-2 levels decreased, indicating enhanced protective effects in both groups, which then resolved. We observed no differences between groups or changes at any time point for ANGPT2, the antagonist of ANGPT1 and Tie-2 binding, which would be indicative of vascular destabilization.

Overall, there were no changes in the inflammatory markers of CRP, TNF-α, IL-1 or IL-6 and no change in endothelial progenitor marker SDF-1, in accordance with results reported by others [16,47]. These results suggest that there were no differences in systemic inflammatory cytokines.

The analyses with classical Framingham-derived cardiovascular risk, which is heavily driven by age, blood pressure, lipid profile, smoking and diabetes status, were not mirrored by single circulating endothelial proteins in this dataset. The absence of significant Framingham–protein correlations (and the lack of Framingham × group interactions) suggests that these vascular biomarkers may capture a pathobiology that is complementary to rather than redundant with respect to traditional risk calculators, including endothelial activation. The consistent inverse trend observed for vWF across both groups warrants cautious interpretation and requires further validation in larger cohorts. The fact that there were no correlations between platelet count and endothelial proteins indicates that platelet number alone does not fully capture potential platelet-driven vascular pathology. In diabetes, the positive trend between platelet count and P-selectin may reflect heightened platelet–endothelial crosstalk.

The STRING analysis of the proteins that changed at baseline showed that there were close associations between them that also extended to proteins that showed changes following iatrogenic hypoglycemia. The fact that P-selectin, PAI-1, E-selectin and cadherin-5 showed large or medium effects sizes according to Cohen’s d suggests that the observed results are likely to be true and not the result of a type 1 error. Functional enrichment analysis demonstrated that this protein network appears to be strongly enriched for Gene Ontology terms related to endothelial activation, leukocyte adhesion, platelet aggregation and impaired fibrinolysis. Central enrichment of cell adhesion, leukocyte migration and hemostasis pathways suggests a coordinated endothelial–immune–thrombotic phenotype. Collectively, these pathways suggest a pro-adhesive, pro-thrombotic endothelial state consistent with chronic low-grade inflammation and endothelial dysfunction.

Given the exploratory nature of this analysis, we reported unadjusted p-values for these baseline results. While FDR correction reduces false positives, it can also mask biologically meaningful effects, as shown by Cohen’s d analysis, in studies with limited sample sizes or correlated variables. Therefore, results that do not survive correction should be interpreted cautiously, but they may still guide hypothesis generation, as shown here. Thus, these exploratory results warrant validation in independent cohorts. The strengths of this study include the fact that the T2D subjects had a relatively short duration of disease, with an average of 4.5 years, and that their diabetes was well controlled by conservative pharmacotherapy of metformin alone. However, this study has several limitations. Metformin improves endothelial dysfunction via the liver kinase B1 (LKB1)/adenosine monophosphate-activated protein kinase (AMPK) pathway [48] and may potentially have affected the baseline and endothelial protein response to hypoglycemia. Given that this study examined plasma proteomics, it cannot be assumed that individual endothelial proteins are consistent with cellular levels or activity. Although clear differences in proteomic endothelial proteins were observed between the T2D and control cohorts, the study is limited by the small size of the study population, and the data needs to be treated with caution, as this was an exploratory hypothesis-generating study. As all subjects were Caucasian, and the results may not be generalizable to other ethnic groups. Adjusting for covariates in our repeated-measures linear model would be preferable; however, our limited sample size constrained our ability to adjust for multiple covariates without compromising model stability and statistical power. Therefore, residual confounding cannot be excluded, and our results should be interpreted with caution.

## 4. Materials and Methods

### 4.1. Study Design

This prospective parallel study enrolled 46 Caucasian participants, comprising 23 adults with T2D and 23 healthy control subjects, as previously described [27]. Recruitment took place at the Diabetes Centre, Hull Royal Infirmary, between 1 March 2017 and 10 January 2018, and written informed consent was obtained from all participants. Control subjects were recruited by advert. Ethical approval was granted by the Northwest–Greater Manchester East Research Ethics Committee (REC number: 16/NW/0518) on 1 February 2017 and the study was registered at ClinicalTrials.gov (NCT03102801) on 6 December 2016. The protocol conformed to the principles of the Declaration of Helsinki. Eligible participants were Caucasian adults aged 40–70 years. For the T2D cohort, inclusion criteria required a disease duration of <10 years, stable treatment with metformin alone for at least 3 months prior to enrolment, glycated hemoglobin (HbA1c) <10% (<86 mmol/mol) and no history of hypoglycemic unawareness or hypoglycemia within the preceding 3 months. In the control group, an oral glucose tolerance test was performed to exclude diabetes. General eligibility criteria for all participants included a body mass index (BMI) of 18–49 kg/m^2^, normal kidney and hepatic function tests, no previous diagnosis of cancer and no contraindications of insulin infusion for the induction of hypoglycemia (including ischemic heart disease, epilepsy, seizures, drop attacks, adrenal insufficiency or treated hypothyroidism).

### 4.2. Biochemical Markers

Blood samples were processed immediately after collection by centrifugation at 2000× *g* for 15 min at 4 °C. Plasma aliquots were prepared and stored at −80 °C within 30 min of collection, pending batch analysis. Biochemical measurements, including fasting plasma glucose (FPG), CRP, total cholesterol, triglycerides, HDL and LDL cholesterol, were performed enzymatically using a Beckman AU 5800 analyzer (Beckman-Coulter, High Wycombe, UK). Platelet count was measured by a Beckman DxH900 (Beckman-Coulter, High Wycombe, UK). Framingham 10-year general risk was calculated according to D’Agostino [49]

### 4.3. Insulin Infusion

Participants underwent an insulin infusion protocol as previously described [27]. Following an overnight fast, intravenous cannulas were placed in both antecubital fossae—one for blood sampling and the other for insulin administration. The infusion of soluble insulin (Humulin S, Lilly, UK) commenced at 08:30 h at an initial rate of 2.5 mU/kg/min, with incremental increases of 2.5 mU/kg/min every 15 min until capillary blood glucose reached ≤2.2 mmol/L (≤40 mg/dL) or a single measurement of ≤2.0 mmol/L (36 mg/dL) with symptoms of hypoglycemia. Prior to induction of hypoglycemia, participants with T2D had their blood glucose stabilized at 5 mmol/L (90 mg/dL). Blood samples were collected at baseline, during hypoglycemia and at predefined time points post hypoglycemia (30 min and 1 h, 2 h, 4 h and 24 h). Once hypoglycemia was confirmed, participants received 150 mL of 10% dextrose intravenously, with capillary glucose monitored every 5 min to ensure recovery. The 24 h post-hypoglycemia sample was obtained the following day.

### 4.4. SOMA-Scan Assay

The SOMAscan assay used to quantify proteins was performed on an in-house Tecan Freedom EVO liquid handling system (Tecan Group, Maennedorf, Switzerland) utilizing buffers and SOMAmers from the SOMAscan HTS Assay 1.3K plasma kit (Somalogic, Boulder, CO, USA) according to the manufacturer’s instructions. Plasma samples were diluted and incubated with streptavidin-coated beads immobilized with dilution-specific SOMAmers, which bind to target proteins via photocleavable linkers. Following washing, bound proteins were biotinylated, released via photocleavage and hybridized onto microarray chips. Fluorescence intensities were measured, and data were normalized using the SOMAscan software pipeline. We used version 3.1 of the SOMAscan Assay, specifically targeting the endothelial biomarker proteins in the SOMAscan panel. Markers of endothelial activation/dysfunction included VEGFA; E-selectin; ICAM1, 2, 3 and 5; VCAM1; P-selectin and cadherin-5. Markers of vascular integrity included ANGPT1 and ANGPT2 and the sTie-2 receptor. Inflammatory markers included TNF-α and pro-inflammatory cytokines (IL1 and IL6). Markers of coagulation and fibrinolysis included TF, tPA, PAI-1, vWF and D-Dimer. SDF-1 was considered as an endothelial progenitor cell-related marker.

### 4.5. STRING Analysis with Functional Enrichment for Gene Ontology

Protein–protein interaction networks were constructed using the STRING database (version 12; https://string-dg.org (accessed on 7 February 2025)) with a minimum interaction confidence score of 0.7. Network topology metrics, including node degree and betweenness centrality, were used to identify key regulatory nodes. Functional enrichment for Gene Ontology (GO; biological process, molecular function and cellular component) and curated pathways (Reactome, KEGG) was performed using a hypergeometric test with Benjamini–Hochberg FDR correction. Enrichment results were integrated with network structure to identify biologically coherent functional modules.

### 4.6. Statistics

No published studies are available that would allow for the performance of a power calculation; therefore, we undertook a hypothesis-generating exploratory pilot study. Power and sample sizes for pilot studies have been reviewed by Birkett and Day [50], who concluded that a minimum of 20 degrees of freedom were required to estimate effect size and variability. Hence, we recruited twenty-three patients to allow for dropouts. To explore initial between-group differences, independent t-tests were applied on normally distributed data, while non-parametric tests (Mann–Whitney U) were applied on data that violated the assumptions of normality when tested using the Kolmogorov–Smirnov test. To explore any potential interactions between group and time, a linear mixed model for repeated measures was run for each endothelial biomarker. Each biomarker was used as the dependent variable, with treatment group, time point and group-by-time interaction included as fixed effects and subject included as a random effect. This approach accounts for individual variation and evaluates both overall trends and group-specific effects. A significant group-by-time interaction would indicate that groups differ in how endothelial biomarker levels change over time. Cohen’s d analysis was undertaken for effect size, and the false discovery rate was applied for multiple testing. Statistical analysis was performed using GraphPad Prism version 10.4.1 (San Diego, CA, USA) and R Studio version 4.4.2.

## 5. Conclusions

In conclusion, baseline endothelial proteins differed between T2D and control subjects, which may suggest local endothelial inflammatory activation leading to a pro-thrombotic, destabilized vascular phenotype characteristic of diabetic vasculopathy. Hypoglycemia may exacerbate this towards a pro-adhesive and pro-thrombotic phenotype, worsening endothelial dysfunction.

## Figures and Tables

**Table 1:** Demographic and clinical characteristics of the study participants [27]. Data are presented as mean ± SD. CRP is presented as median (interquartile range).

Baseline	T2D (*n* = 23)	Controls (*n* = 23)	*p*-Value
Age (years)	64 ± 8	60 ± 10	0.015
Sex (M/F)	12/11	11/12	0.77
Weight (kg)	90.9 ± 11.1	79.5 ± 8.8	<0.0001
Height (cm)	167 ± 14	169 ± 5	0.64
BMI (kg/m^2^)	32 ± 4	28 ± 3	<0.0001
Systolic BP (mmHg)	132 ± 8	122 ± 8	0.001
Diastolic BP (mmHg)	81 ± 7	75 ± 6	0.003
Duration of diabetes (years)	4.5 ± 2.2	N/A	
HbA1c (mmol/mol)	51.2 ± 11.4	37.2 ± 2.2	<0.0001
HbA1c (%)	6.8 ± 1.0	5.6 ± 0.2	<0.0001
Total cholesterol (mmol/L)	4.2 ± 1.0	4.8 ± 0.77	0.014
Triglycerides (mmol/L)	1.7 ± 0.7	1.34 ± 0.6	0.055
HDL-cholesterol (mmol/L)	1.1 ± 0.3	1.5 ± 0.4	0.001
LDL-cholesterol (mmol/L)	2.23 ± 0.8	2.7 ± 0.87	0.051
CRP (mg/L)	1.91 (2.28)	2.1 (1.15)	0.33
Platelet count (10^−9^/L)	272 ± 50	240 ± 50	0.13
Framingham score (%)	25.8 ± 9.4	12.6 ± 5.7	1.3 × 10^−6^

BMI, body mass index; BP, blood pressure; HbA1c, HemoglobinA1c; HDL, high-density lipoprotein; LDL, low-density lipoprotein; CRP, C-reactive protein; N/A, not applicable.

**Table 2:** Plasma levels of endothelial cell proteins at baseline in control and type 2 diabetes (T2D) subjects with changes at baseline or during the hypoglycemia time course. A linear mixed model for repeated measures was used to analyze changes over time while accounting for individual differences. This method allows us to see both overall trends and group-specific effects over multiple time points. The inclusion of the group x time interaction tests whether the way the two groups change over time differs or not. An interaction that is not statistically significant means that, while there may be differences between the groups (diabetes vs. control) and changes over time, the ways the two groups change over time are not significantly different from each other. Levels of proteins are reported as Relative Fluorescent Units (RFU) and mean (SD).

Protein	Control_Mean ± SD	Diabetes_Mean ± SD	Cohen_d	CI_Lower	CI_Upper	*p*_Value	Effect Size
P-Selectin	11,567 ± 2655	15,415 ± 4483	−1.04	−1.66	−0.43	0.0001	Large
PAI-1	1196 ± 911	2127 ± 1228	−0.86	−1.47	−0.26	0.01	Large
E-Selectin	24,728 ± 7418	30,439 ± 9222	−0.68	−1.28	−0.09	0.03	Medium
Cadherin-5	11,611 ± 1758	9983 ± 2598	0.73	0.14	1.33	0.02	Medium
ANGPT1	433 ± 156	752 ± 610	−0.72	−1.31	−0.12	0.02	Medium
ICAM3	594 ± 203	554 ± 88	0.26	−0.32	0.84	0.39	Small
ICAM5	814 ± 220	723 ± 209	0.42	−0.16	1.01	0.16	Small
sTie-2	1661 ± 337	1525 ± 349	0.40	−0.19	0.98	0.18	Small

Intercellular adhesion molecule-3, 5 (ICAM3, 5); angiopoietin-1(ANGPT1); soluble angiopoietin-1 receptor (sTie-2); plasminogen activator inhibitor-1 (PAI-1; Serpine 1).

## Data Availability

The original contributions presented in this study are included in the article/Supplementary Materials. Further inquiries can be directed to the corresponding author.

## Supplementary Materials

The following supporting information can be downloaded at: https://www.mdpi.com/article/10.3390/ijms27020822/s1.

## Abbreviations

The following abbreviations are used in this manuscript:

T2D	Type 2 diabetes
PAI-1; serpine 1	Plasminogen activator inhibitor-1
ANGPT1	Angiopoietin-1
sTie-2	Soluble angiopoietin-1 receptor
ICAM	Intercellular adhesion molecule
VCAM1	Vascular cell adhesion molecule-1
ANGPT2	Angiopoietin 2
IL	Interleukin
SDF-1	Stromal cell-derived factor-1
vWF	von Willebrand factor
VEFGA	Vascular endothelial growth factor
TNFα	Tumor necrosis factor alpha
tPA	Tissue plasminogen activator
TF	Tissue factor
Nrf2	Nuclear Factor Erythroid 2-Related Factor 2
u-PA	Urokinase-type plasminogen activator
Tie-2	Angiopoietin-1 receptor
PI3K	Phosphatidylinositol 3-kinase
Akt	Protein kinase B
p38 MAPK	p38 mitogen-activated protein kinase
LKB1	Liver kinase B1
GenAI	Generative artificial intelligence
AMPK	Adenosine monophosphate-activated protein kinase
HbA1c	HaemoglobinA1c
BMI	Body mass index
BP	Blood pressure
HDL	High-density lipoprotein
LDL	Low-density lipoprotein
CRP	C-reactive protein
RFU	Relative Fluorescent Units
