# Supplementary material for: Iatrogenic Hypoglycemia in Type 2 Diabetes Affects Endothelial Proteins Involved in Cardiovascular Dysfunction
Source: Int J Mol Sci. 2026 Jan 14;27(2):822. doi: 10.3390/ijms27020822 (PMC12841412; doi:10.3390/ijms27020822)
Supplement: Supplementary file 1 [file ijms-27-00822-s001.zip › ijms-4031116-supplementary.pdf]

Figure S1: Plasma protein markers that showed no changes at any timepoint during the study. Blood sampling was performed at baseline (BL), at hypoglycemia (0 min) and post-hypoglycemia (30 min, 1, 2, 4 and 24 h) for controls (black circles) and for type 2 diabetes (T2D) (blue squares). At BL, blood glucose (BG) was 7.5 ± 0.4 mM (for T2D) and 5.0 ± 0.1 mM (for control, C). At point of hypoglycemia, blood glucose (BG) was 2.0 ± 0.03 mM (for T2D) and 1.8 ± 0.05 mM (for control). A, intercellular adhesion molecule-1 (ICAM1); B, vascular cell adhesion molecule-1 (VCAM1); C, von Willebrand factor (vWF); D, ICAM2; E, vascular endothelial growth factor (VEGFA); F, angiopoietin 2 (ANGPT2). Data are presented as mean ± SEM.

Figure S2: Plasma protein markers that showed no changes at any timepoint during the study. Blood sampling was performed at baseline (BL), at hypoglycemia (0 min) and post-hypoglycemia (30 min, 1, 2, 4 and 24 h) for controls (black circles) and for type 2 diabetes (T2D) (blue squares). At BL, blood glucose (BG) was 7.5 ± 0.4 mM (for T2D) and 5.0 ± 0.1 mM (for control, C). At point of hypoglycemia, blood glucose (BG) was 2.0 ± 0.03 mM (for T2D) and 1.8 ± 0.05 mM (for control). A, tumor necrosis factor alpha (TNFalpha); B, tissue factor (TF); C, interleukin 6 (IL6); D, D-dimer; E, tissue plasminogen activator (tPA); F, stromal cell-derived factor-1 (SDF-1); G, IL1a. Data are presented as mean ± SEM.

Table S1: Plasma levels of endothelial cell proteins at baseline in Control and Type 2 Diabetes (T2D) subjects with no changes at baseline or during the hypoglycemia time course. A linear mixed model for repeated measures was used to analyze changes over time while accounting for individual differences. This method allows us to see both overall trends and group-specific effects over multiple time points. The inclusion of the interaction, group x time, tests whether the way the two groups change over time differs or not. When the interaction is not statistically significant, it means that, while there may be differences between the groups (diabetes vs. control) and changes over time, the way the two groups change over time is not significantly different from each other. Levels of proteins are in Relative Fluorescent Units (RFU); Mean (SD).

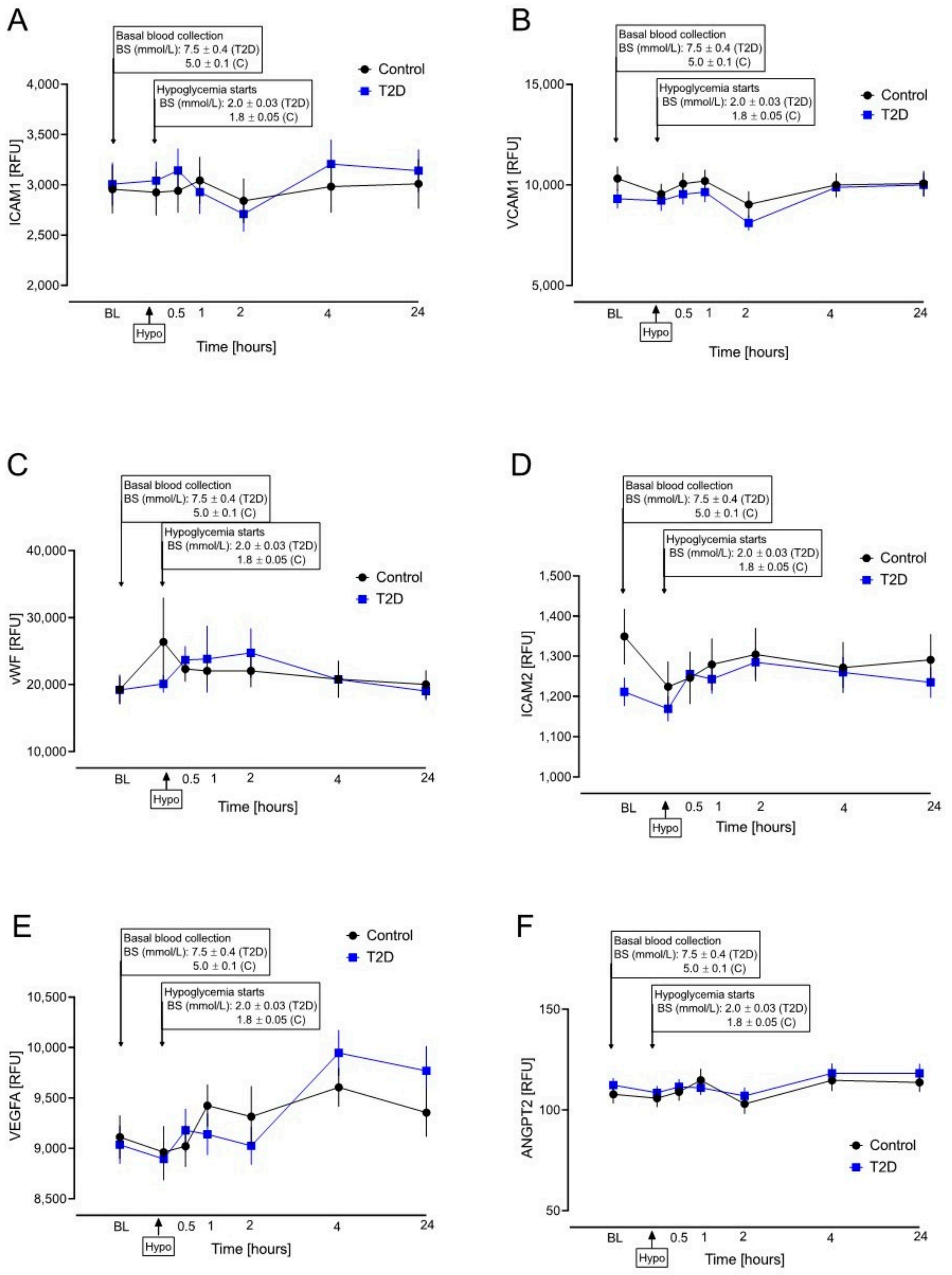
A
Basal blood collection
BS (mmol/L): 7.5 ± 0.4 (T2D)
5.0 ± 0.1 (C)
Hypoglycemia starts
BS (mmol/L): 2.0 ± 0.03 (T2D)
1.8 ± 0.05 (C)
Control
T2D
ICAM1 [RFU]
4,000
3,500
3,000
2,500
2,000
BL
Hypo
0.5
1
2
4
24
Time [hours]
B
Basal blood collection
BS (mmol/L): 7.5 ± 0.4 (T2D)
5.0 ± 0.1 (C)
Hypoglycemia starts
BS (mmol/L): 2.0 ± 0.03 (T2D)
1.8 ± 0.05 (C)
Control
T2D
VCAM1 [RFU]
15,000
10,000
5,000
BL
Hypo
0.5
1
2
4
24
Time [hours]
C
Basal blood collection
BS (mmol/L): 7.5 ± 0.4 (T2D)
5.0 ± 0.1 (C)
Hypoglycemia starts
BS (mmol/L): 2.0 ± 0.03 (T2D)
1.8 ± 0.05 (C)
Control
T2D
vWF [RFU]
40,000
30,000
20,000
10,000
BL
Hypo
0.5
1
2
4
24
Time [hours]
D
Basal blood collection
BS (mmol/L): 7.5 ± 0.4 (T2D)
5.0 ± 0.1 (C)
Hypoglycemia starts
BS (mmol/L): 2.0 ± 0.03 (T2D)
1.8 ± 0.05 (C)
Control
T2D
ICAM2 [RFU]
1,500
1,400
1,300
1,200
1,100
1,000
BL
Hypo
0.5
1
2
4
24
Time [hours]
E
Basal blood collection
BS (mmol/L): 7.5 ± 0.4 (T2D)
5.0 ± 0.1 (C)
Hypoglycemia starts
BS (mmol/L): 2.0 ± 0.03 (T2D)
1.8 ± 0.05 (C)
Control
T2D
VEGFA [RFU]
10,500
10,000
9,500
9,000
8,500
BL
Hypo
0.5
1
2
4
24
Time [hours]
F
Basal blood collection
BS (mmol/L): 7.5 ± 0.4 (T2D)
5.0 ± 0.1 (C)
Hypoglycemia starts
BS (mmol/L): 2.0 ± 0.03 (T2D)
1.8 ± 0.05 (C)
Control
T2D
ANGPT2 [RFU]
150
100
50
BL
Hypo
0.5
1
2
4
24
Time [hours]

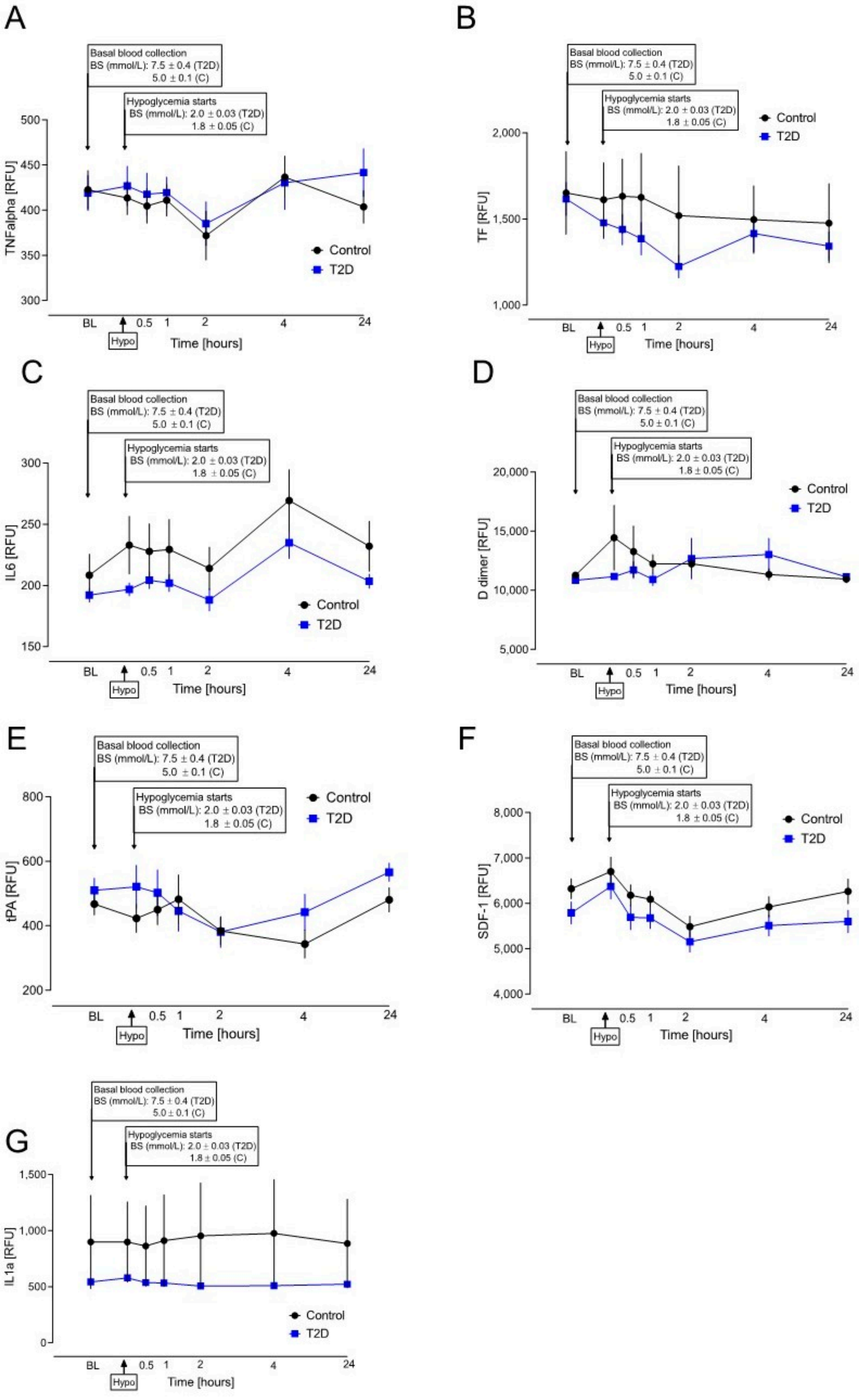
A
Basal blood collection
BS (mmol/L): 7.5 ± 0.4 (T2D)
5.0 ± 0.1 (C)
Hypoglycemia starts
BS (mmol/L): 2.0 ± 0.03 (T2D)
1.8 ± 0.05 (C)
TNFalpha [RFU]
Control
T2D
BL
Hypo
0.5
1
2
4
24
Time [hours]
B
TF [RFU]
C
IL6 [RFU]
D
D dimer [RFU]
E
tPA [RFU]
F
SDF-1 [RFU]
G
IL1a [RFU]

Protein	Control_mean±SD	Diabetes_mean±SD	Cohen_d	CI_lower	CI_upper	p_value	Effect size
ICAM2	1349 ± 330	1211 ± 165	0.53	-0.06	1.11	0.08	Medium
VCAM1	10321 ± 2822	9308 ± 2224	0.40	-0.19	0.98	0.18	Small
ANGPT-2	108 ± 21	112 ± 16	-0.24	-0.82	0.34	0.42	Small
IL1a	900 ± 1998	543 ± 111	0.25	-0.33	0.83	0.40	Small
IL6	208 ± 83	192 ± 28	0.26	-0.32	0.84	0.38	Small
tPA	467 ± 161	510 ± 182	-0.25	-0.83	0.33	0.41	Small
D-dimer	11279 ± 1386	10856 ± 1224	0.32	-0.26	0.91	0.28	Small
SDF-1	6323 ± 1065	5791 ± 1170	0.48	-0.11	1.06	0.11	Small
ICAM1	2957 ± 1145	3007 ± 1014	-0.05	-0.62	0.53	0.88	Negligible
vWF	19246 ± 10446	19193 ± 9392	0.01	-0.57	0.58	0.99	Negligible
VEGFA	9112 ± 1017	9037 ± 901	0.08	-0.50	0.66	0.79	Negligible
TNFα	423 ± 102	419 ± 93	0.04	-0.54	0.62	0.90	Negligible
TF	1652 ± 1154	1617 ± 462	0.04	-0.54	0.62	0.89	Negligible

Intercellular adhesion molecule-1 (ICAM1); vascular cell adhesion molecule-1 (VCAM1); vascular endothelial growth factor (VEGFA); angiopoietin 2 (ANGPT2); tumor necrosis factor alpha (TNFα); interleukin 1, 6 (IL1, 6); tissue factor (TF); tissue plasminogen activator (tPA); von Willebrand factor (vWF); stromal cell-derived factor-1 (SDF-1)
